# Polygenic risk score is a predictor of adenomatous polyps at screening colonoscopy

**DOI:** 10.1186/s12876-021-01645-4

**Published:** 2021-02-12

**Authors:** Michael J. Northcutt, Zhuqing Shi, Michael Zijlstra, Ayush Shah, Siqun Zheng, Eugene F. Yen, Omar Khan, Mohammad Imran Beig, Polina Imas, Adam Vanderloo, Obaid Ansari, Jianfeng Xu, Jay L. Goldstein

**Affiliations:** 1 Department of Internal Medicine, University of Chicago Medicine, NorthShore University HealthSystem, 2650 Ridge Ave, Evanston, IL 60201 USA; 2 Division of Gastroenterology, University of Chicago Medicine, NorthShore University HealthSystem, 2650 Ridge Ave, Evanston, IL 60201 USA; 3 Program for Personalized Cancer Care, NorthShore University HealthSystem, 1001 University Place, 1001 University Place, Evanston, IL 60201 USA; 4 Department of Clinical Analytics and Health Information Technology, NorthShore University HealthSystem, 4901 Searle Parkway, Skokie, IL 60076 USA; 5 Chicago, IL 60647 USA

**Keywords:** Colonoscopy, Adenomatous polyps, Polygenic risk score, GRS, Colorectal cancer, Screening

## Abstract

**Background:**

Single nucleotide polymorphism (SNP)-based polygenic risk scoring is predictive of colorectal cancer (CRC) risk. However, few studies have investigated the association of genetic risk score (GRS) with detection of adenomatous polyps at screening colonoscopy.

**Methods:**

We randomly selected 1769 Caucasian subjects who underwent screening colonoscopy from the Genomic Health Initiative (GHI), a biobank of NorthShore University HealthSystem. Outcomes from initial screening colonoscopy were recorded. Twenty-two CRC risk-associated SNPs were obtained from the Affymetrix™ SNP array and used to calculate an odds ratio (OR)-weighted and population-standardized GRS. Subjects with GRS of < 0.5, 0.5–1.5, and > 1.5 were categorized as low, average and elevated risk.

**Results:**

Among 1,769 subjects, 520 (29%) had 1 or more adenomatous polyps. GRS was significantly higher in subjects with adenomatous polyps than those without; mean (95% confidence interval) was 1.02 (1.00–1.05) and 0.97 (0.95–0.99), respectively, *p* < 0.001. The association remained significant after adjusting for age, gender, body mass index, and family history, *p* < 0.001. The detection rate of adenomatous polyps was 10.8%, 29.0% and 39.7% in subjects with low, average and elevated GRS, respectively, *p*-trend < 0.001. Higher GRS was also associated with early age diagnosis of adenomatous polyps, *p* < 0.001. In contrast, positive family history was not associated with risk and age of adenomatous polyps.

**Conclusions:**

GRS was significantly associated with adenomatous polyps in subjects undergoing screening colonoscopy. This result may help in stratifying average risk patients and facilitating personalized colonoscopy screening strategies.

## Background

National guidelines recommend that, in the absence of known risk factors, patients considered to be average risk for developing colorectal cancer (CRC) should begin screening at age 50 years [1–4]. The recommendation for CRC screening with colonoscopy is based on a meaningful reduction in incidence and mortality from CRC offered by timely screening [5]. The combined gastroenterology society guidelines recognize risk factors including a family history of CRC (single first-degree relative with CRC or advanced adenoma diagnosed at age < 60 years or two first-degree relatives with CRC or advanced adenomas), personal history of inflammatory bowel disease, and/or a personal or family history of a hereditary colorectal cancer syndrome [6]. For patients with any of the above risk factors, colonoscopy is initiated at an earlier age and/or with more frequent follow up exams than those patients at average risk for CRC [2]. While CRC incidence has declined steadily over the past two decades in the population aged 50 years and older, limited demographic-based risk factors currently in use inadequately predict heightened CRC risk regardless of age.

Moving our screening from population-based risk to individual risk requires the incorporation of genetic predisposition. ﻿Many CRC risk-associated single-nucleotide polymorphisms (SNPs) have been identified and confirmed from genome-wide association studies [7–17]. Although the effect of individual SNPs is modest, there is a stronger cumulative effect. SNP-based Genetic Risk Score (GRS), an odds ratio (OR)-weighted and population-standardized polygenic risk score derived from well-established CRC risk–associated SNPs, has been consistently associated with CRC risk [18–23]. In brief, it is calculated by multiplying the per-allele OR for each SNP and normalizing the risk by the mean risk expected in the population. Such an approach has been successfully implemented for other malignancies in predicting risk for breast cancer, prostate cancer, CRC and other cancers [24, 25]. Few studies to date have tested the association between GRS and screening-detected adenomatous polyps [26]. As the detection of adenomatous polyps is the primary purpose of screening colonoscopy, the aim of this study is to test the association of GRS with risk of adenomatous polyps in a study population undergoing screening colonoscopy [27]. We hypothesize that, for patients who underwent screening colonoscopy at our institution and were diagnosed with advanced adenomas and non-advanced adenomas, CRC-risk associated SNPs can be used to construct a GRS to more accurately identify an individual’s risk for developing adenomatous polyps than current screening recommendations.

## Methods

We requested the medical record number of 200,000 patients who underwent colonoscopy between January 1, 2006 and September 5, 2018 at NorthShore University HealthSystem, a large community-based academic healthcare system in northern Illinois, involving four suburban hospitals. These subjects were screened for inclusion in the Genomic Health Initiative (GHI), a DNA biobank of the NorthShore University HealthSystem. Consistent with prior studies focused on GRS, we aimed to simulate an average risk population [20]. Thus,

patients must have also met the following inclusion criteria: (1) no prior or current diagnosis of CRC, hereditary CRC syndrome, ulcerative colitis or Crohn’s disease, (2) underwent screening colonoscopy at NorthShore, (3) age > 45 years old at the first screening colonoscopy, (4) self-reported Caucasian, and (5) with available genotyping data from the Affymetrix Axiom™ Biobank Plus Genotyping Array. Our study only included Caucasians due to insufficient sample size for other ancestry groups. Accordingly, 1769 patients met the inclusion criteria and detailed clinical and demographic information from each patient’s index screening colonoscopy including age, gender, BMI, family history, indication for colonoscopy as well as colonoscopy outcomes from the first screening colonoscopy were extracted from the electronic medical record. Location, size, and histologic characteristics of polyps were recorded. Positive colonoscopy was defined as any adenomatous polyps per study protocol. Advanced adenomas were defined as adenomatous polyps greater than or equal to 1 cm in size, or with a “villous” component (tubulovillous or villous), or with foci of high grade dysplasia. The study was approved by the internal review board of NorthShore University Health System.

Genotypes of 22 known CRC risk-associated SNPs were extracted from a customized Axiom™ Biobank Plus Genotyping Array (Additional file: Table 1). These SNPs were identified from evidence-based review of literature and met the following criteria: (1) discovered from genome-wide association studies of CRC in Caucasian subjects, with at least 1000 cases and 1000 controls in the first stage; (2) confirmed in additional stages with combined *P* < 5 × 10^–8^; and (3) independent, with linkage disequilibrium (LD) measurement (*r*^2^ < 0.2) between any pair of SNPs [7–17].

**Table 1:** Association of variables with screening colonoscopy outcomes

			Univariate analysis		Multivariable analysis	
Variables	Positive^1^ (N = 520)	Negative^2^ (N = 1249)	OR (95% CI)	*p*	OR (95% CI)	*p*
Age at colonoscopy, mean (95% CI)	61.0 (60.3–61.6)	60.2 (59.8–60.6)	1.01 (1–1.03)	0.04	1.01 (1–1.02)	0.1
Gender, No. (%) of male	247 (47.5)	411 (32.9)	1.84 (1.55–2.2)	< 0.001	1.8 (1.51–2.15)	< 0.001
Positive family history, No. (%)	40 (7.7)	87 (7.0)	1.11 (0.8–1.54)	0.66	1.14 (0.81–1.58)	0.53
BMI, mean (95% CI)	29.5 (28.9–30)	28.0 (27.7–28.3)	1.04 (1.02–1.05)	< 0.001	1.04 (1.02–1.05)	< 0.001
GRS, mean (95% CI)	1.02 (1–1.05)	0.97 (0.95–0.99)	1.61 (1.24–2.08)	< 0.001	1.63 (1.25–2.12)	< 0.001

^1^Positive: adenomatous polyps

^2^Negative: no polyps or hyperplastic polyps

*CI* confidence interval, *OR* odds ratio, *BMI* body mass index; GRS, genetic risk score

Genetic risk score (GRS), an established odds ratio (OR)-weighted and population-standardized polygenic risk score was computed for each subject based on the 22 CRC risk-associated SNPs [24]. Briefly, GRS was calculated by multiplying the per-allele OR for each SNP and normalizing the risk by the average risk expected in the population (w)

$$\begin{aligned} & {\text{GRS}} = \mathop \prod \limits_{i = 1}^{n} \frac{{OR_{i}^{{g_{i} }} }}{{W_{i} }} \\ & W_{i} = f_{i}^{2} OR_{i}^{2} + 2f_{i} \left( {1 - f_{i} } \right)OR_{i} + (1 - f_{i} )^{2} \\ \end{aligned}$$

where, *g*_i_ stands for the genotype of SNP *i* for an individual (0, 1, or 2 risk alleles, respectively), *OR*_*i*_ stands for the OR of SNP *i* and *f*_*i*_ stands for the risk allele frequency of SNP *i*. Allelic ORs obtained from the external studies and allele frequencies in the gnomAD (Non-Finnish European [NFE] population) were used in the calculation. Because GRS is population-standardized, its mean is expected to be 1.0 and its values can be interpreted as relative risk to the general population. As such, subjects with GRS of < 0.5, 0.5–1.5 and > 1.5 were categorized as low, average and elevated risk prior to analysis.

Both univariable and multivariable analyses were performed. For univariable analysis, differences of quantitative variables and qualitative variables among groups were tested using T-test and Chi-square, respectively. Multivariable analyses were performed to test independent effects of predictors using logistic regression modeling. A Kaplan–Meier adenomatous polyp diagnosis-free survival analysis was used to test association between GRS and age at abnormal colonoscopy. Statistical analyses were performed by R version 3.5.2, and two-tailed *P* < 0.05 was considered statistically significant.

## Results

Among the 1,769 subjects included in this study, 520 subjects had one or more adenomatous polyps on their first screening colonoscopy, yielding an adenoma detection rate of 29.4% overall (24.6% for females, 37.5% for males). While male gender and higher body mass index (BMI) were significantly associated with risk of adenomatous polyps at screening colonoscopy in univariable and multivariable analysis, age was significant on only univariable analysis (Table 1). The mean and 95% confidence interval (CI) age at first screening colonoscopy was 61.0 (60.3–61.6) and 60.2 (59.8–60.6) years old, respectively, in subjects with and without adenomatous polyps, OR (95%CI) = 1.01 (1.00–1.03)*, p* = 0.04. The mean (95% CI) BMI was 29.5 (29.0–30.0) and 28.0 (27.7–28.3), respectively, in subjects with and without adenomatous polyps [OR (95%CI) = 1.04 (1.02–1.05), *p* < 0.001]. The proportion of male gender was 47.5% and 32.9%, respectively in subjects with and without adenomatous polyps [OR (95%CI) = 1.84 (1.55–2.20), *p* < 0.001]. In contrast, positive family history was not associated with risk of having adenomatous polyps; it was found in 7.7% and 7.0% subjects with and without adenomatous polyps [OR (95%CI) = 1.11 (0.8–1.54), *p* = 0.7].

**Fig. 1:**
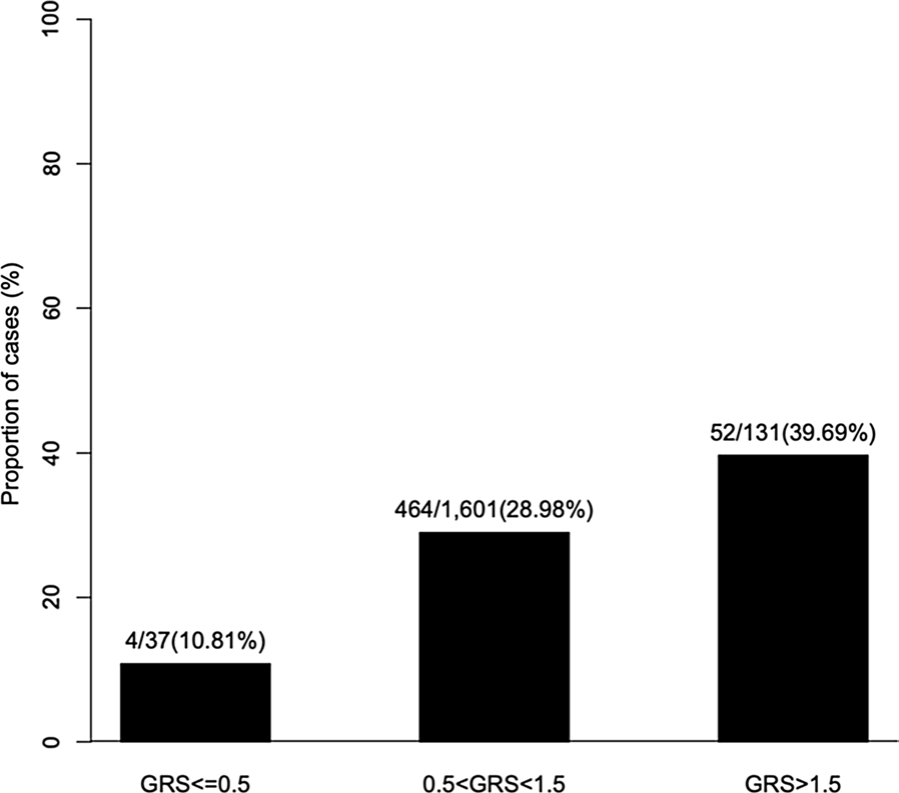
Detection rates of adenomatous polyps using GRS for 22 known risk-associated SNPs. Incidence of adenomatous polyps varies directly with increasing GRS. *p*-trend < 0.001

Higher GRS was significantly associated with increased risk of adenomatous polyps. The mean (95% CI) GRS was 1.02 (1.00–1.05) and 0.97 (0.95–0.99), respectively, in subjects with and without adenomatous polyps [OR (95%CI) = 1.61 (1.24–2.08), *p* = 0.003]. This association was independent of other known predictors in a multivariable analysis; OR (95%) = 1.63 (1.25–2.12), *p* = 0.003 when adjusting for age, gender, BMI and family history (Table 1). The detection rate of adenomatous polyps increased with higher categorical GRS risk groups, 10.8%, 29.0% and 39.7% in subjects with low, average and elevated GRS risk group, respectively, *p*-trend < 0.001 (Fig. 1).

**Fig. 2:**
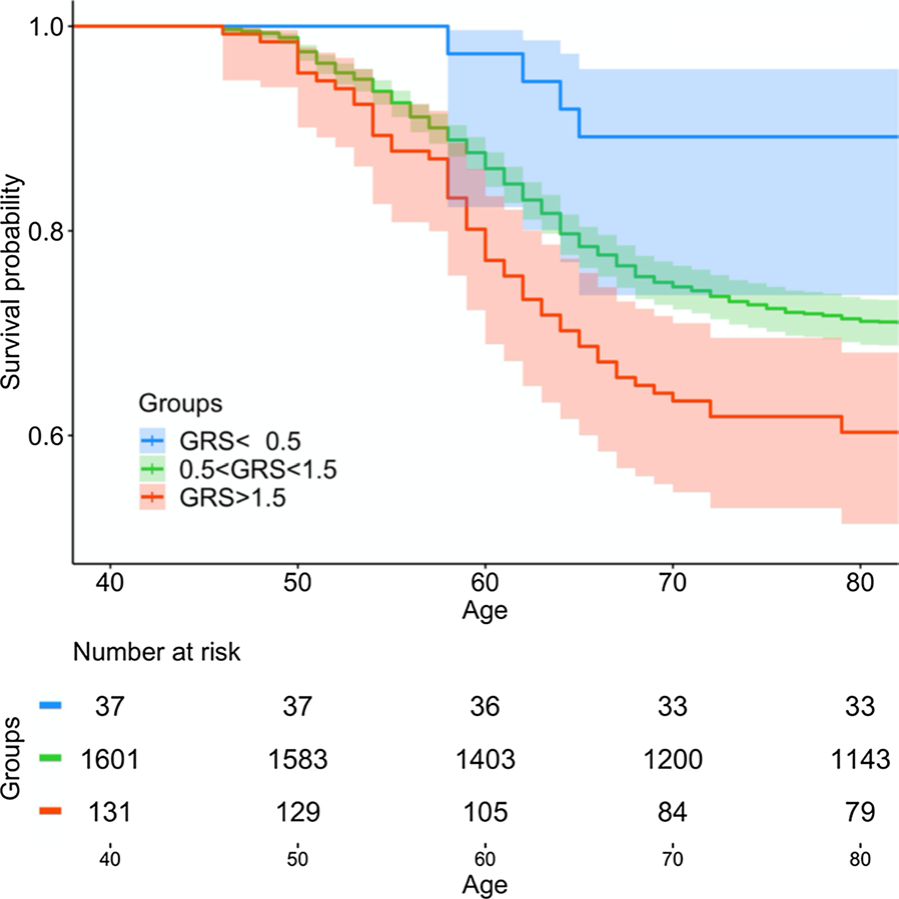
Kaplan–Meier analysis demonstrating subjects in the higher GRS risk groups had an earlier age diagnosis of adenomatous polyp(s). *p* < 0.001

A Kaplan–Meier curve analysis examining the time to the first diagnosis of adenomatous polyps over the lifespan of the patient starting at birth (time = zero) revealed that subjects in the higher GRS risk groups had an earlier age diagnosis of adenomatous polyp(s). The difference was statistically significant based on the Log rank test, *p* = 0.001 (Fig. 2).

Association of GRS with detailed colonoscopy outcomes is presented in Table 2. The mean (95% CI) of GRS was similar for subjects with different sizes of adenomatous polyps; 1.02 (0.97–1.06) and 1.03 (1.00–1.07) for those with size < 0.5 and ≥ 0.5 cm, respectively. The association was significant in patients with adenomatous polyps > 0.5 cm (*p* < 0.001). Likewise, the mean (95% CI) of GRS was similar for subjects with different numbers of adenomatous polyps; 1.04 (1.00–1.07) and 1.01 (0.96–1.15) for those with one or more than one adenomatous polyps, respectively. The mean GRS was also similar for adenomatous polyps at different locations (cecum/ascending colon, transverse/descending colon, or rectum/sigmoid). The association was significant in patients for polyps in the cecum/ascending colon (*p* = 0.02). The mean GRS (95% CI) was 1.02 (0.97–1.08) for 126 subjects with advanced adenoma(s), but did not reach statistical significance (*p* = 0.1).

**Table 2:** Association of GRS with detailed features of colonoscopy outcomes

Variables	No. subjects	GRS, mean (95% CI)	OR (95% CI)	*p*
No polyps or HP	1249	0.97 (0.95–0.99)	1	
Adenomatous polyps	520	1.02 (1–1.05)	1.61 (1.24–2.08)	< 0.001
Polyp size < 0.5 cm	203	1.02 (0.97–1.06)	1.49 (1.03–2.13)	0.07
Polyp size ≥ 0.5 cm	316	1.03 (1–1.07)	1.68 (1.23–2.27)	< 0.001
Advanced adenoma	126	1.02 (0.97–1.08)	1.56 (0.99–2.42)	0.10
Total no. of polyps				
No. = 1	304	1.04 (1–1.07)	1.77 (1.3–2.41)	< 0.001
No. > 1	216	1.01 (0.96–1.15)	1.38 (0.96–1.96)	0.14
Location				
CA	296	1.02 (0.98–1.06)	1.56 (1.14–2.12)	0.02
TD	197	1.02 (0.97–1.06)	1.49 (1.02–2.14)	0.08
RS	159	1.02 (0.97–1.08)	1.57 (1.05–2.33)	0.06

*GRS* genetic risk score, *CI* confidence interval, *OR* odds ratio, *HP* hyperplastic polyp(s), *BMI* body mass index, *CA* cecum/ascending colon, *TD* transverse/descending colon, *RS* rectum and sigmoid colon

## Discussion

In this retrospective analysis of screening colonoscopy data from 1769 patients at a community-based, high volume healthcare system involving four hospital-based gastroenterology labs and two free-standing endoscopy units, we developed and characterized a SNP-based, odds ratio-weighted and population-standardized polygenic genetic risk score (GRS). Our results demonstrated the GRS was significantly higher for patients who had adenomatous polyps on colonoscopy compared to those who did not (Table 1). Furthermore, this novel association was independent of age, gender, BMI and family history on multivariable analysis. As shown in Fig. 1, the proportion of cases with adenomatous polyps trended up in patients stratified by three tertiles of GRS, which is similar to previous studies [20, 22].

Based on our findings, higher GRS risk groups have an earlier diagnosis of adenomatous polyps (Fig. 2). Kaplan–Meier time-to-event analysis from birth (time = zero) to first screening colonoscopy identifying an adenomatous polyp(s) differed significantly among the three GRS risk groups based on the Log rank test. The time-to-event analysis is justified for germline predictors such as GRS because they can be measured at age zero. Since the probability of finding a polyp increases with age, this analysis demonstrates the predictive nature for adenomatous polyps across GRS risks at any given age. Furthermore, in order to reconcile the possibility that the lower GRS group simply includes older subjects when they underwent first colonoscopy, we calculated mean age at colonoscopy for the three GRS groups: 59.0, 60.4, and 60.6 years for subjects with low, intermediate and high GRS with *p* = value 0.51. Therefore, this observation is unlikely driven by age at first colonoscopy.

Furthermore, the predictive value of GRS for adenomatous polyps versus no adenomatous polyps was primarily driven by polyps measuring greater than or equal to 0.5 cm (Table 2). For polyps > 1 cm (i.e. advanced adenomas), our data shows a trend toward similar predictive value which is consistent with prior studies [22]. The lack of statistical significance is presumably related to small sample size; only 126 of the total 1769 patients had advanced adenomas in our study population. Table 2 also suggests that GRS was predictive of a single polyp, but not of multiple polyps. Thus, higher GRS increases the susceptibility to initiation of any polyps (any number, size and/or location). Likewise, as seen with other types of cancer, GRS may therefore be associated with susceptibility to cancer, not the aggressiveness of cancer [24, 25].

Interestingly, GRS for adenomatous polyps localized to the cecum and ascending colon was significantly higher than for patients with no adenomatous polyps on colonoscopy. These results parallel the prior study by Weigl et al., in which odds ratios were largest for proximal advanced neoplasms [22]. As our data incorporates all right sided polyps, including sessile serrated polyps, and is consistent with other studies, we believe this highlights the potential future clinical utility of GRS for risk stratification. Specifically, right sided CRC confers higher morbidity and mortality with poorer outcomes, and any strategy to identify these patients earlier would have a greater clinical impact [28].

The clinical importance of detecting and removing small nonadvanced adenomas is currently controversial. However, our data for adenomatous polyps < 1 cm demonstrated that detection of these lesions may have clinical relevance by further stratifying risk for an individual in our population. It is noteworthy that the most recent guidelines have lengthened the interval to follow up colonoscopy from 5–10 years to 7–10 years in patients with one to two small adenomas < 10 mm in size [29]. Our data, however, lends support to maintaining more conservative surveillance intervals in patients with nonadvanced lesions.

In addition, our study confirms previously identified CRC risk factors by demonstrating male gender and higher BMI were significantly associated with risk of adenomatous polyps on multivariable analysis. Interestingly, family history was not associated with presence of adenomatous polyps. This finding reinforces the limited clinical utility of family history alone as a CRC risk factor [20]. In our medical record, as in many other healthcare systems, family history is variably recorded and often incomplete. This may be an issue of misreporting among family members as to their health-related conditions, incomplete documentation by healthcare providers, and/or the true lack of predictive power of family history. As such, this data collected by chart review adds a great deal of subjectivity. In contrast, the objective nature of genetic stratification leads to a more individualized and reproducible approach.

Despite the objectivity that genetic data provides to any individual, there are inherent limitations of utilizing a SNP-based approach for risk stratification. As in other studies, our investigation used high impact, well-established risk-associated SNPs for calculation of GRS (Additional file: Table 1) [20, 22]. GRS calculations are based on the number of SNPs tested on the chosen array which may be limited by commercial and research availability. Thus, our GRS only incorporates the known common genetic susceptibility variants for CRC that were included in the customized Axiom™ Biobank Plus Genotyping Array. It is intuitive that with further identification of SNPs associated with CRC that the predictive value of a GRS will further be improved.

In contrast to other studies that tested associations of GRS with CRC risk, our study is the first one to test the association of GRS with risk of adenomatous polyps among average-risk subjects from a community-based health care system [20]. However, as a single healthcare system, our data is limited in number and scope compared to studies using large epidemiological consortia. As our study only included Caucasians based on the demographics of the population at the different sites of our medical centers, there is potential selection bias and generalizability of our results should be limited to the Caucasian population. Furthermore, with the lack of access to additional risk factors (i.e. physical activity, alcohol/tobacco use, and dietary factors such as low fiber and high red meat consumption) and limited numbers of patients compared to these population-based studies, it is not possible to unite genetic data with epidemiologic data to develop a truly integrated GRS. Specifically, in contrast to the study by Jeon et al.which used two large population-based data consortia, we did not strive to reproduce their findings[20]. Despite these limitations, our results were similar to studies with access to such data.

An additional consideration is that we included patients who had a coded indication (ICD-10 code) for screening colonoscopy. However, this may not always reflect the true indication for the procedure as stated; embedded in this population may be symptomatic patients, thus introducing a higher probability of neoplastic disease. Although not evaluated, we believe that this theoretical selection bias would minimally affect our results.

## Conclusions

An ideal population-based CRC screening program would include a predictable and reproducible stratification schema to appropriately initiate and surveil patients. While current screening guidelines rely on demographics and epidemiologic risk factors for CRC, the availability of known or suspected risk factors, and the reproducibility of that data, may be lacking. A reasonable objective is to focus on genetic predisposition to stratify large populations of patients. With the option of genetic testing for patients becoming more accepted, and with the continued identification of additional SNPs associated with CRC, GRS can be more easily utilized for a patient at the bedside. Specifically, our GRS for detection of adenomatous colon polyps could be combined with known risk factors for CRC to support clinical decision making for an individualized approach to CRC screening. Genetic risk scoring for other malignancies, including breast, prostate and ovarian cancers, has proven to have substantial clinical utility [24, 25]. Our study, along with others, initiates the possibility of refining and expanding this approach for population-based risk identification to determine optimal screening strategies.

## Supplementary Information

**Additional file 1:** **SupplementaryTable 1.** Known risk-associated SNPs for colorectal canceravailable from Affymetrix Axiom™Biobank Plus Genotyping Array.

## Data Availability

The datasets used and/or analysed during the current study are available from the corresponding author on reasonable request.
