# Supplementary material for: Polygenic risk score is a predictor of adenomatous polyps at screening colonoscopy
Source: BMC Gastroenterol. 2021 Feb 12;21:65. doi: 10.1186/s12876-021-01645-4 (PMC7881602; doi:10.1186/s12876-021-01645-4)
Supplement: Supplementary file 1 — Additional file 1: SupplementaryTable 1. Known risk-associated SNPs for colorectal canceravailable from Affymetrix Axiom™Biobank Plus Genotyping Array. [file 12876_2021_1645_MOESM1_ESM.docx]

| **Supplementary Table 1.** Known risk-associated SNPs for colorectal cancer available from Affymetrix Axiom™ Biobank Plus Genotyping Array | | | | | | |
| --- | --- | --- | --- | --- | --- | --- |
| **SNP** | **Chromosome** | **Position** | **Risk allele** | **Risk allele frequency** | **Odds ratio** | **Reference** |
| rs6691170 | 1 | 222045446 | T | 0.339787 | 1.06 | Houlston RS.Nat Genet.2010 |
| rs10936599 | 3 | 169492101 | C | 0.779708 | 1.08 | Houlston RS.Nat Genet.2010 |
| rs647161 | 5 | 134499092 | A | 0.618072 | 1.07 | Jia WH,Nat Genet. 2013 |
| rs16892766 | 8 | 117630683 | C | 0.093778 | 1.25 | Tomlinson IP.Nat Genet.2008 |
| rs3824999 | 11 | 74345550 | G | 0.395611 | 1.20 | Whiffin N,Hum Mol Genet. 2014 |
| rs3802842 | 11 | 111171709 | C | 0.297764 | 1.15 | Tenesa A.Nat Genet.2008 |
| rs10774214 | 12 | 4368352 | T | 0.459643 | 1.04 | Jia WH,Nat Genet. 2013 |
| rs7136702 | 12 | 50880216 | T | 0.408275 | 1.06 | Houlston RS.Nat Genet.2010 |
| rs11169552 | 12 | 51155663 | C | 0.749553 | 1.09 | Houlston RS.Nat Genet.2010 |
| rs1957636 | 14 | 54560018 | T | 0.516293 | 1.08 | Tomlinson IP,PLoS Genet. 2011 |
| rs4779584 | 15 | 32994756 | T | 0.341785 | 1.15 | Tomlinson IP,PLoS Genet. 2011 |
| rs9929218 | 16 | 68820946 | G | 0.72056 | 1.10 | COGENT Study,Nat Genet.2008 |
| rs4939827 | 18 | 46453463 | T | 0.353235 | 1.18 | Broderick P,Nat Genet. 2007 |
| rs10411210 | 19 | 33532300 | C | 0.795237 | 1.15 | COGENT Study,Nat Genet.2008 |
| rs961253 | 20 | 6404281 | A | 0.32519 | 1.12 | Tomlinson IP,PLoS Genet. 2011 |
| rs4925386 | 20 | 60921044 | C | 0.577073 | 1.08 | Houlston RS.Nat Genet.2010 |
| rs10795668 | 10 | 8701219 | G | 0.757007 | 1.12 | Tomlinson IP.Nat Genet.2008 |
| rs6687758 | 1 | 222164948 | G | 0.210459 | 1.09 | Schmit SL,J Natl Cancer Inst. 2018 |
| rs4444235 | 14 | 54410919 | C | 0.415885 | 1.08 | Schmit SL,J Natl Cancer Inst. 2018 |
| rs4813802 | 20 | 6699595 | G | 0.282094 | 1.08 | Schmit SL,J Natl Cancer Inst. 2018 |
| rs11903757 | 2 | 192587204 | C | 0.16506 | 1.16 | Peters U.Gastroenterology.2012 |
| rs5934683 | X | 9751474 | C | 0.511889 | 1.07 | Dunlop MG.Nat Genet.2012 |
|  |  |  |  |  |  |  |
| 1: Dunlop MG, Dobbins SE, Farrington SM, et al. Common variation near CDKN1A, POLD3 and SHROOM2 influences colorectal cancer risk. Nat Genet. 2012 May 27;44(7):770-6. | | | | | | |
| 2: Peters U, Jiao S, Schumacher FR, et al. Identification of Genetic Susceptibility Loci for Colorectal Tumors in a Genome-Wide Meta-analysis. Gastroenterology. 2013 Apr;144(4):799-807. | | | | | | |
| 3: Houlston RS, Cheadle J, Dobbins SE, et al. Meta-analysis of three genome-wide association studies identifies susceptibility loci for colorectal cancer at 1q41, 3q26.2, 12q13.13 and 20q13.33. Nat Genet. 2010 Nov;42(11):973-7. | | | | | | |
| 4: Schmit SL, Edlund CK, Schumacher FR, et al. Novel Common Genetic Susceptibility Loci for Colorectal Cancer. J Natl Cancer Inst. 2019 Feb 1;111(2):146-157. | | | | | | |
| 5: Broderick P, Carvajal-Carmona L, Pittman AM, et al. A genome-wide association study shows that common alleles of SMAD7 influence colorectal cancer risk. Nat Genet. 2007 Nov;39(11):1315-7. | | | | | | |
| 6: COGENT Study, Houlston RS, Webb E, et al. Meta-analysis of genome-wide association data identifies four new susceptibility loci for colorectal cancer. Nat Genet. 2008 Dec;40(12):1426-35. | | | | | | |
| 7: Jia WH, Zhang B, Matsuo K, et al. Genome-wide association analyses in East Asians identify new susceptibility loci for colorectal cancer. Nat Genet. 2013 Feb;45(2):191-6. | | | | | | |
| 8: Tenesa A, Farrington SM, Prendergast JG, et al. Genome-wide association scan identifies a colorectal cancer susceptibility locus on 11q23 and replicates risk loci at 8q24 and 18q21. Nat Genet. 2008 May;40(5):631-7. | | | | | | |
| 9: Tomlinson IP, Carvajal-Carmona LG, Dobbins SE, et al. Multiple common susceptibility variants near BMP pathway loci GREM1, BMP4, and BMP2 explain part of the missing heritability of colorectal cancer. PLoS Genet. 2011 Jun;7(6):e1002105. | | | | | | |
| 10: Tomlinson IP, Webb E, Carvajal-Carmona L, et al. A genome-wide association study identifies colorectal cancer susceptibility loci on chromosomes 10p14 and 8q23.3. Nat Genet. 2008 May;40(5):623-30. | | | | | | |
| 11: Whiffin N, Hosking FJ, Farrington SM, et al. Identification of susceptibility loci for colorectal cancer in a genome-wide meta-analysis. Hum Mol Genet. 2014 Sep 1;23(17):4729-37. | | | | | | |
